# Ultra-long-term efficacy and safety of catheter-based renal denervation in resistant hypertension: 10-year follow-up outcomes

**DOI:** 10.1007/s00392-024-02417-2

**Published:** 2024-03-07

**Authors:** Hussam Al Ghorani, Saarraaken Kulenthiran, Lucas Lauder, Michael Johannes Maria Recktenwald, Juliane Dederer, Michael Kunz, Felix Götzinger, Sebastian Ewen, Christian Ukena, Michael Böhm, Felix Mahfoud

**Affiliations:** 1 Klinik für Innere Medizin III, Kardiologie, Angiologie und Internistische Intensivmedizin, Universitätsklinikum des Saarlandes, Saarland University, Kirrberger Str. 100, Gebäude 41, 66421 Homburg/Saar, Germany; 2 Medizinische Klinik II – Kardiologie/Angiologie, Marien Hospital Herne – Universitätsklinikum der Ruhr Universität Bochum, Hölkeskampring 40, 44625 Herne, Germany

**Keywords:** Resistant hypertension, Office blood pressure, 24-h ambulatory blood pressure monitoring, Renal function, Safety

## Abstract

**Background:**

Randomized sham-controlled trials have confirmed the efficacy and safety of catheter-based renal denervation in hypertension. Data on the very long-term effects of renal denervation are scarce.

**Aims:**

This study evaluates the 10-year safety and efficacy of renal denervation in resistant hypertension.

**Methods:**

This prospective single-center study included patients with resistant hypertension undergoing radio-frequency renal denervation between 2010 and 2012. Office blood pressure, 24-h ambulatory blood pressure, antihypertensive medication, color duplex sonography, and renal function were assessed after 1-, 2- and 10-years.

**Results:**

Thirty-nine patients completed the 10-year follow-up (mean follow-up duration 9.4 ± 0.7 years). Baseline office and 24-h ambulatory systolic blood pressure were 164 ± 23 mmHg and 153 ± 16 mmHg, respectively. After 10 years, 24-h ambulatory and office systolic blood pressure were reduced by 16 ± 17 mmHg (*P* < 0.001) and 14 ± 23 mmHg (*P* = 0.001), respectively. The number of antihypertensive drugs remained unchanged from 4.9 ± 1.4 to 4.5 ± 1.2 drugs (*P* = 0.087). The estimated glomerular filtration rate declined within the expected range from 69 (95% CI 63 to 74) to 60 mL/min/1.73m^2^ (95% CI 53 to 68; *P* < 0.001) through 10-year follow-up. Three renal artery interventions were documented for progression of pre-existing renal artery stenosis in two patients and one patient with new-onset renal artery stenosis. No other adverse events were observed during the follow-up.

**Conclusion:**

Renal denervation was safe and sustainedly reduced ambulatory and office blood pressure out to 10 years in patients with resistant hypertension.

**Graphical abstract:**

Left panel, Change in 24-h and office SBP. Right panel, eGFR over time. SBP, systolic blood pressure; eGFR, estimated glomerular filtration rate.

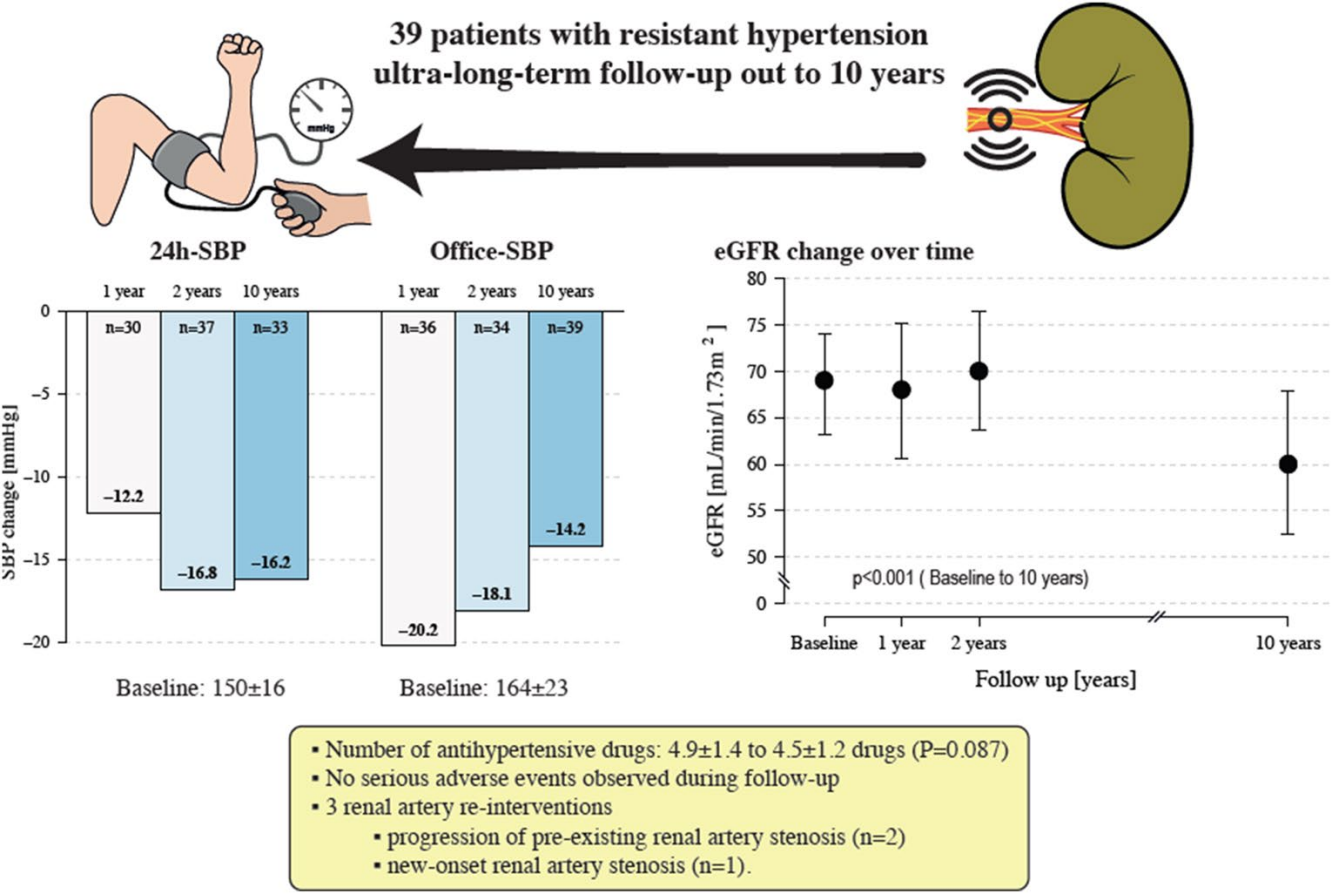

**Supplementary Information:**

The online version contains supplementary material available at 10.1007/s00392-024-02417-2.

## Introduction

Despite the availability of various effective treatment modalities such as lifestyle modification and antihypertensive drugs, a significant proportion of patients remains above guideline-recommended blood pressure (BP) treatment targets [1–3]. The kidneys, with their sympathetic innervation, play a crucial role in BP regulation [4]. Against this background, device-based therapies to modulate the activity of the autonomic nervous system have been investigated [5]. Catheter-based renal denervation (RDN) represents a minimally-invasive treatment for uncontrolled hypertension, which uses radiofrequency (RF), ultrasound, or perivascular injection of alcohol to target the perivascular sympathetic nerve fibers surrounding the renal arteries [5]. Several randomized, sham-controlled trials [6–10] and meta-analyses [11, 12] demonstrated the efficacy and safety of RDN in patients with and without concomitant antihypertensive therapy [13]. Long-term data from the Global Symplicity Registry and the randomized, sham-controlled SPYRAL-ON MED and RADIANCE-HTN SOLO trials demonstrated both the safety and efficacy of the procedure with significant and sustained office and ambulatory BP reductions out to 3 years [14–17]. We recently published the 10-year long-term data on 24-h (24-h) ambulatory BP (ABP) [18]. This study sought to scrutinize the i) BP-lowering efficacy on both office and 24-h ABP, ii) changes in renal resistance indices (RRI), and iii) safety of RF-RDN in resistant hypertension at long-term follow-up of 10 years.

## Methods

### Study design

The study design and 24-h ABP outcomes have been published elsewhere [18]. Patients with resistant hypertension and with no changes in medication for a minimum of 2 weeks before enrolment were included in this prospective, single-arm, single-center study.

The inclusion criteria were:

1. Adult patients aged ≥ 18 years with resistant hypertension (office systolic BP (SBP) ≥ 140 mmHg and/or diastolic BP (DBP) ≥ 90 mmHg, despite treatment with ≥ 3 antihypertensive drugs (including a diuretic) at maximally tolerated doses) [2]
2. Eligibility for RDN as defined by the instructions for use of the Symplicity RDN system (Medtronic, Inc, Santa Rosa, CA)

The exclusion criteria were:

1. eGFR of < 45 mL/min/1.73 m^2^
2. Known secondary cause of hypertension other than sleep apnea or chronic kidney disease (CKD).

All patients gave written informed consent and were treated between August 2010 and October 2012. Optimization of antihypertensive therapy was considered in all patients before RDN. Medical history data was obtained from patient records, and the comorbidities were defined according to current European guidelines. Patients were asked about whether they had taken their medication at defined doses. Treating physicians and patients were instructed not be adjusted without consulting the study center beforehand. The study was performed at the Saarland University Hospital, and the local ethic committee approved the study (Symplicity Extension, NCT01888315). The trial complies with the Declaration of Helsinki.

### Procedure and follow-up data

RDN was performed by all patients using the RF-based Symplicity Flex single-electrode catheter system (Medtronic, Santa Rosa, CA, USA) by experienced operators. The RF-ablations were performed in the main renal artery without branch treatment. Follow-up data were collected for the present study at baseline, after one year, two years, and 10 (±0.5) years post-RDN. At each visit, investigators performed a medical history, physical examination, and documented changes in hypertension therapy. At all study time points, the importance of lifestyle modification and medication adherence was emphasized. The office BP was measured three times using an automated oscillometric device (Omron HEM-705 monitor, Omron Healthcare, Vernon Hills, Illinois, USA) with the patient sitting quietly for at least five minutes with one to two minutes between each measurement. Office BP was considered as the mean of the last two readings. 24-h ABP monitoring was performed with an automated oscillometric device (Spacelabs 90207, Spacelabs Healthcare, Snoqualmie, Washington, USA) before the procedure and at each follow-up visit. Duplex sonography was performed to assess renal artery integrity. RRI was also measured via duplex sonography as an estimate of renal perfusion. Intrarenal Doppler spectra were obtained at 6 representative locations (2 in the cranial, 2 in the middle, and 2 in the caudal third of the kidney) of the interlobar arteries along the border of medullary pyramids in each kidney. Peak systolic (V_max_) and end-diastolic velocity (V_min_) were obtained, and the dimensionless renal resistive index (RRI) was calculated as RRI = (peak systolic velocity—end-diastolic velocity) / peak systolic velocity. The mean RRI was calculated using 6 measurements from each kidney. Both velocities in the renal arteries were measured in the origin and the proximal, middle, and distal segments of each renal artery. The eGFR was calculated using the Chronic Kidney Disease Epidemiology Collaboration (CKD-EPI) formula [19].

### Efficacy and safety objectives

This study aimed to assess the efficacy and safety of RDN through 10 years of follow-up. The outcomes of interest were: change in mean 24-h ABP, office BP, and the number of antihypertensive drugs. Safety endpoints were incidence of periprocedural and long-term adverse events (i.e. bleeding, dissection, pseudoaneurysm at the femoral access, onset of renal artery stenosis or re-intervention). Changes in renal function were measured by eGFR and RRI.

### Statistical analysis

Continuous variables were presented as means ± standard deviation. Categorical variables were presented as counts and percentages and were compared with McNemar’s test. Within-group differences in continuous variables from baseline to follow-up were tested with the paired t-test and repeated measures ANOVA using mixed model. A two-tailed *p*-value < 0.05 was considered statistically significant. Statistical analyses were done with IBM SPSS Statistics (version 27.0; SPSS Inc., Chicago, Illinois, USA). Missing data were not imputed.

## Results

### Baseline and procedural characteristics

A total of 39 patients were included (supplementary material). The mean follow-up duration was 9.4 ± 0.7 years. Patients were 62 ± 8 years of age, mostly male (64%), with a mean body mass index of 32 ± 5 kg/m^2^. The most prevalent comorbidities were a history of heart failure (46%), left ventricular hypertrophy (44%), and type 2 diabetes (44%) (Table 1). Although patients were treated with a mean of 4.9 ± 1.4 antihypertensive drugs, 24-h ABP and office BP at baseline were 152 ± 16/85 ± 14 mmHg and 164 ± 23/91 ± 13, respectively, with a mean ambulatory heart rate of 64 ± 9 bpm. The procedural characteristics are depicted in Table 2.

**Table 1:** Baseline characteristics

Characteristics	Value (*n* = 39)
Male	25 (64.1%)
Age, years	62 ± 8
Body mass index, kg/m^2^	32 ± 5
Diabetes Type 2	17 (43.6%)
Left ventricular hypertrophy*	17 (43.6%)
eGFR, ml/min/1.73 m^2^ eGFR < 60 ml/min/1.73 m^2^	68.7 ± 16.6 12 (30.8%)
Currently smoking	3 (7.7%)
Obstructive sleep apnea	10 (25.6%)
Coronary artery disease	7 (17.9%)
Atrial fibrillation	7 (17.9%)
History of renal artery stenosis	3 (7.7%)
History of heart failure (HFpEF)	18 (46.2%)
History of myocardial infarction	2 (5.1%)
Office systolic blood pressure, mmHg	164 ± 23
Office diastolic blood pressure, mmHg	91 ± 13
24-h systolic blood pressure, mmHg	152 ± 16
24-h diastolic blood pressure, mmHg	85 ± 14

Results are shown as n (%) or mean ± standard deviation. eGFR, estimated glomerular filtration rate, HFpEF: heart failure with preserved ejection fraction, *according to the history of patient or by echocardiography according to the left ventricular mass index

**Table 2:** Procedural characteristics

Characteristics	Value (*n* = 39)
Total number of ablations	10.2 ± 2.3
Contrast (ml)	85.7 ± 43.0
Procedural duration (minutes)	78.8 ± 20.3

Values are means ± standard deviation

### Efficacy outcomes

**Fig. 1:**
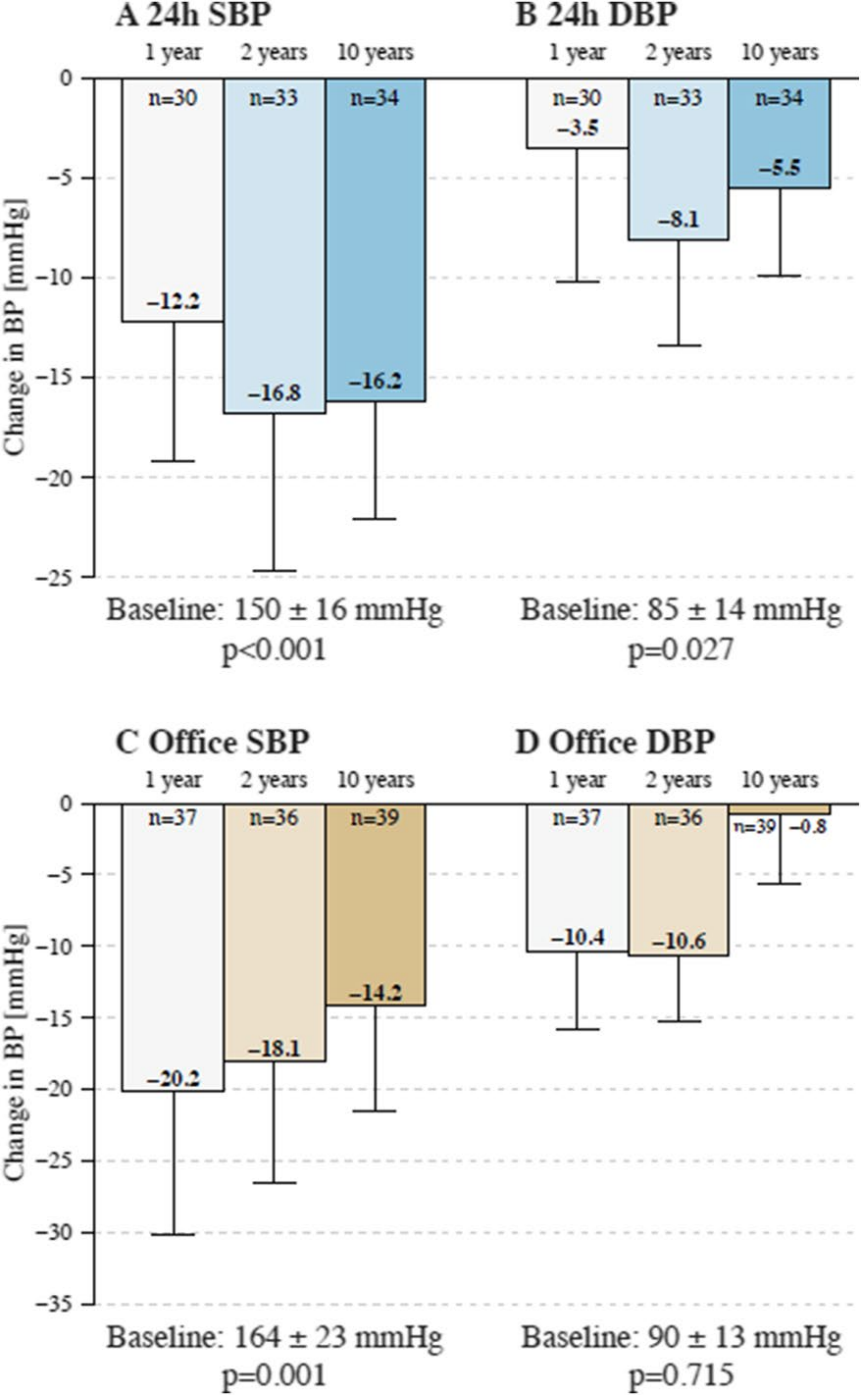
Blood pressure changes. (A) Change in mean 24-h systolic, (B) 24-h diastolic, (C) office systolic and (D) office diastolic blood pressure at 1 year, 2 years, and 10 years follow-up after renal denervation. Error bars represent 95% confidence intervals. The p-values were obtained from a repeated measures ANOVA test with a linear-mixed model

**Fig. 2:**
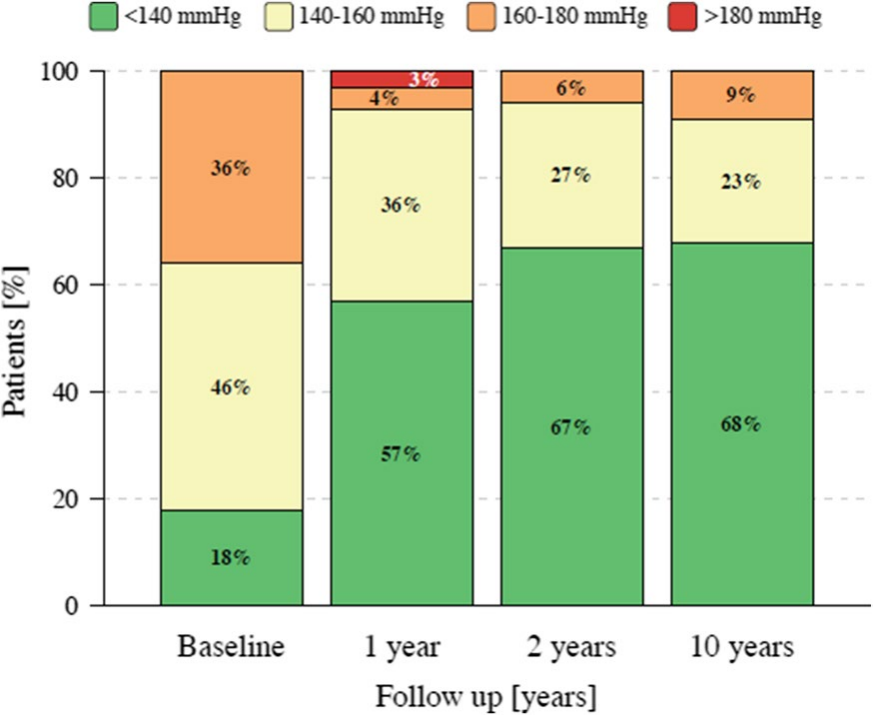
Hypertension severity according to 24-h systolic blood pressure**.** Proportion of patients with 24-h SBP < 140 mmHg at 1 year, 2 years, and 10 years follow-up. Colors or ranges does not represent a recommendation of target blood pressure values. SBP: systolic blood pressure

The changes in BP during follow-up are summarized in Table 3. At 1 year, 24-h ABP decreased by -12 ± 19/-4 ± 18 mmHg. This decrease was sustained through 10 years of follow-up (Fig. 1). At 10-year follow-up, 24-h SBP and DBP decreased by -16 ± 17 mmHg and -6 ± 13 mmHg (*P* < 0.001 and 0.027) (Fig. 1A, B), respectively [18]. The office SBP decreased by -20 ± 30 mmHg at 1 year, -18 ± 25 mmHg at 2 years, and -14 ± 23 mmHg at 10 years (P for all = 0.001) (Fig. 1C), respectively. The office DBP significantly decreased by -10 ± 16 at 1 year and -11 ± 14 mmHg at 2 years (P for both < 0.001). At 10 years follow-up, no significant reduction in office DBP was observed (-1 ± 15 mmHg; *P* = 0.700 for baseline vs. 10 years) (Fig. 1D). The proportion of patients with 24-h SBP < 140 mmHg increased from 17.9% at baseline to 56.7%, 66.7% and 67.7%, at 1 year, 2 years, and 10 years, respectively (Fig. 2). The mean number of antihypertensive drugs did not change significantly (4.9 ± 1.4 at baseline to 4.5 ± 1.2 at 10 years, *P* = 0.087) (Table 4).

**Table 3:** Changes in blood pressure and estimated glomerular filtration rate from baseline over time

	Baseline (n)	1 year follow-up (n)	2 years follow-up (n)	10 years follow-up (n)	*P*-value^*^
Office SBP, mmHg	164.0 ± 23.0 (39)	144.4 ± 33.0 (37)	145.3 ± 22.3 (36)	149.8 ± 20.1 (39)	< 0.001
Office DBP, mmHg	90.5 ± 13.3 (39)	80.2 ± 15.5 (37)	80.0 ± 12.2 (36)	89.6 ± 12.6 (39)	0.715
24-h SBP, mmHg	152.5 ± 16.1 (39)	138.3 ± 16.1 (30)	135.6 ± 16.0 (33)	134.2 ± 16.3 (34)	0.001
24-h DBP, mmHg	85.3 ± 13.5 (39)	80.0 ± 15.6 (30)	77.2 ± 11.1 (33)	79.3 ± 12.1 (34)	0.027
eGFR, ml/min/1.73m^2^	68.7 ± 16.6 (39)	68.0 ± 21.9 (37)	70.2 ± 19.1 (36)	60.2 ± 23.8 (39)	< 0.001
RRI	0.71 ± 0.07 (39)	0.68 ± 0.07 (37)	0.68 ± 0.07 (36)	0.70 ± 0.10 (37)	0.186

Values are means ± standard deviation. ^*^Repeated measures ANOVA with linear mixed models. *DBP,* diastolic blood pressure; *eGFR,* estimated glomerular filtration rate; *RRI,* renal resistive index; *SBP,* systolic blood pressure

**Table 4:** Antihypertensive medication at baseline and long-term follow-up

	Baseline (*n* = 39)	10 years follow-up (*n* = 39)	*P*-value^*^
Number of antihypertensive drugs	4.92 ± 1.4	4.54 ± 1.2	0.087
ACE inhibitors and angiotensin receptor blockers	100.0%	97.5%	0.899
Beta-blockers	89.7%	82.1%	0.375
Calcium channel blockers	69.2%	82.1%	0.180
Diuretics	76.9%	89.7%	0.180
Aldosterone antagonists	12.8%	33.3%	0.021
Alpha-adrenergic blockers	12.8%	5.1%	0.453
Direct-acting vasodilators	25.6%	7.7%	0.039
Alpha-2 receptor agonist	41.0%	30.8%	0.344
Direct renin inhibitors	48.7%	0.0%	1.0

Values are means ± standard deviations and percentages. ^*^10 years follow-up vs. baseline using McNemar test for categorical variables, paired *t*-test for number of antihypertensive medication classes. *ACE,* Angiotensin-converting-enzyme

### Safety outcomes

**Fig. 3:**
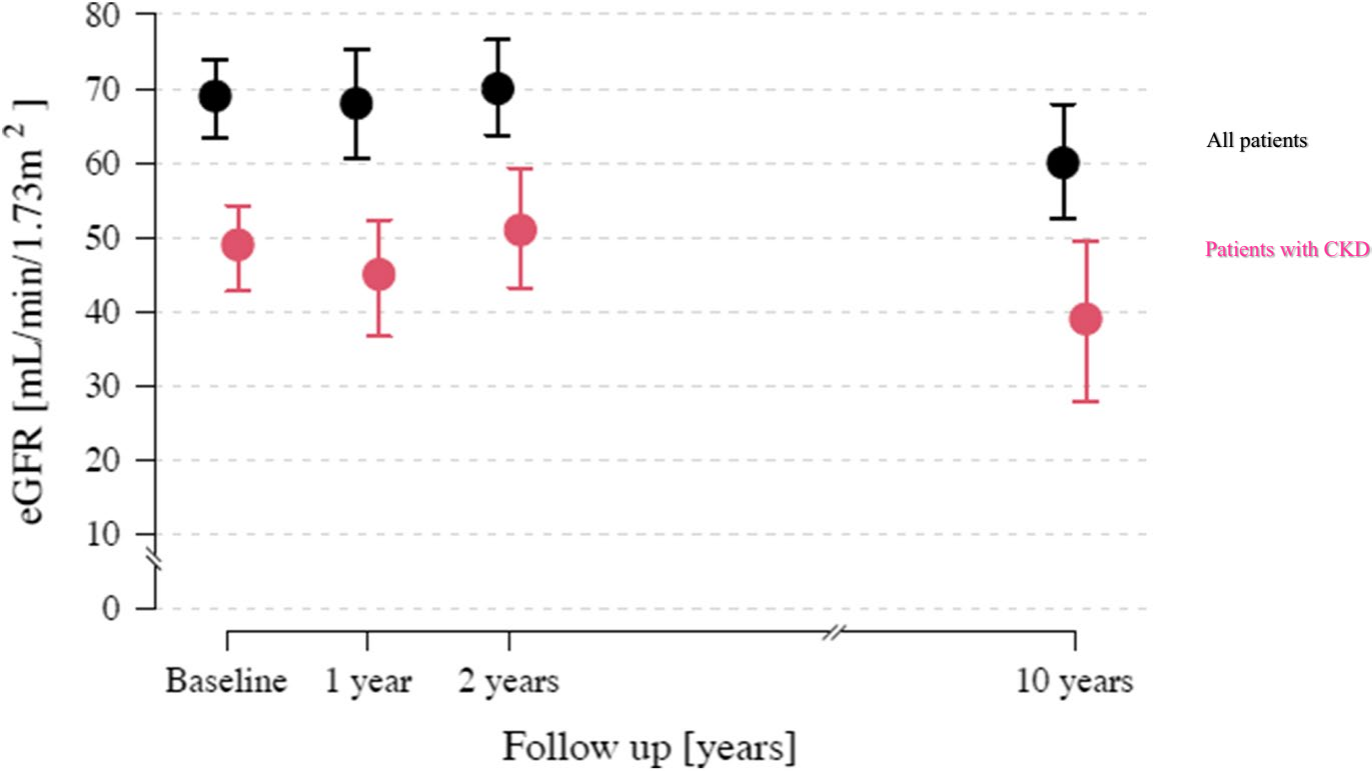
Renal function over time. Values are mean the estimated glomerular filtration rates (eGFR) over time in all patients and in patients (*n* = 12) with CKD at baseline (eGFR < 60 mL/min/1.73 m^2^). Error bars represent 95% confidence intervals. eGFR: estimated glomerular filtration rate

At 1 and 2 years, the eGFR remained unchanged compared with baseline but declined significantly between baseline and 10 years from 69 (95% CI 63 to 74.) to 60 (95% CI 53 to 70;) ml/min/1.73 m^2^ (*P* < 0.001 for change from baseline to 10 years) (Fig. 3). The proportion of patients with an eGFR < 60 ml/min/1.73 m^2^ was 31% at baseline and increased to 56% at long-term follow-up. There were no cases of doubling in serum creatinine or end-stage renal disease. RRI declined significantly from 0.71 ± 0.07 to 0.68 ± 0.07 at 1 and 2 years but remained stable at 10 years ( 0.70 ± 0.10 at 10 years (*P* = 0.186) (Table 3), respectively.

No severe peri-procedural adverse events were documented. In one case, renal artery vasospasm occurred during RF-energy delivery, which resolved directly without further sequelae. During long-term follow-up, there was a progression of pre-existing renal artery stenosis in two out of three previously diagnosed patients, which was treated with stent implantation after 1 and 10 years, respectively. New-onset renal artery stenosis was diagnosed in one subject after 1 year, which was treated with a drug-coated balloon.

## Discussion

Clinical trials and real-world registries have confirmed a significant BP reduction following RDN out to 3 years [14–17]. Recently, two studies assessed the long-term outcomes of RDN in patients with resistant hypertension and showed sustained reductions in 24-h BP up to 10 years post-RDN [18–20]. However, further data on the very long-term efficacy and safety, including office BP, BP control rates, utilization of antihypertensive drugs, and RRI, have not yet been reported. Herein we analyzed the efficacy, using both ABP and standardized office BP measurements, and safety following catheter-based RF-RDN using laboratory and sonographic parameters through 10 years of follow-up. We observed sustained reductions in BP after RDN with a favorable short- and long-term safety profile. RRI decreased significantly, and more importantly, renal function as assessed by eGFR did not decline beyond the expected range for patients with resistant hypertension [21].

Recently published sham-controlled trials using RF [6, 7] and ultrasound [8–10, 17] technologies have proven the efficacy of RDN in patients with and without concomitant antihypertensive medication up to 3 years [6–10, 17]. These trials allowed up-titration of antihypertensive medications after assessing the primary outcome at 2 [8–10] and 3 months post-RDN [7], which often makes it challenging to attribute the full BP-lowering effect to the RDN procedure or the changes in medication. The efficacy and safety of RDN documented herein adds relevant information about the procedure in real-world patients with resistant hypertension at long-term follow-up, since clinical data on this respect are scarce [14]. An analysis of 3.077 patients showed a comparable BP decrease in 24-h ABP and office BP at 3 years of follow-up [14]. Herein, 24-h ABP was primarily used to assess the efficacy of RDN, which has been shown to associate stronger with cardiovascular risk than office BP [22, 23]. Thus, achieving BP control in 24-h ABP reduces mortality and improves cardiovascular outcomes (cardiovascular mortality, non-fatal cardiovascular events, heart failure, and stroke) [22, 23]. In this cohort with resistant hypertension, the proportion of patients with a 24-h SBP of  < 140 mmHg increased from 17.9% at baseline to 56.7%, 66.7%, and 67.7% at 1 year, 2 years, and 10 years, respectively (Fig. 2). Notably, the number of antihypertensive drugs were numerically but insignificantly reduced over the follow-up period indicating that the observed BP changes were unlikely related to increase in drug treatment. Recently, a post-hoc analysis from the SPYRAL HTN-OFF MED Pivotal trial [24] demonstrated that patients in the RDN group were less likely to experience hypertensive urgencies (SBP ≥ 180 mmHg) and other safety concerns that required immediate use of anti-hypertensive medications compared to the sham control group [24].

Preclinical data in normotensive and hypertensive sheep suggest that renal nerves can regrow after RDN with variable restoration of functional responses in long-term follow-up [25, 26]. The observed BP reductions herein were sustained until ten years after RDN, which indicates that even if a potential structural nerve regrow following the procedure has occurred, as suggested in preclinical studies [27], it may not be relevant for BP control. This is in line with data from the Global Symplicity Registry [14] and clinical trials, [16, 17, 28, 29] which also showed a durable, significant BP reduction out to 36 months. These data are in line with a recently published study showing that RDN resulted in significant and robust reductions in both office and ambulatory systolic and diastolic BP [20]. In our study only office DBP reached baseline values at 10 years after a significant reduction at 1 and 2 years. This observation could be a coincidence. Further possible explanations for this observation are the natural progression of hypertension with a progressive increase in BP.

The favorable long-term safety profile post procedure observed herein reiterates the safety outcomes derived from randomized, sham-controlled clinical trials [6–10, 15–17, 28, 29], meta-analysis [30], and real-world registries [14, 18, 20, 31]. The eGFR remained unchanged in the first 2 years and declined by -9 ml/min/1.73 m^2^ at the very long-term follow-up. This was associated with an increase in the proportion of patients with CKD stage 3 (from 30.8 to 56.4%). One has to keep in mind that the decline in eGFR with age depends on the level of BP and the presence of comorbidities [32–35]. The annual decline in patients with severe uncontrolled hypertension has been reported to range between -0.5 and -6 mL/min/1.73 m^2^ [32–35]. Thus, the eGFR decline in the population studied herein was less than expected, indicating that RDN did not impair kidney function but, in contrast, might have attenuated the GFR decrease in these patients at high cardiovascular risk.

RRI has been associated with progression of renal impairment, as well as morbidity and mortality in hypertensive patients [36, 37]. There was no significant change in RRI through 10 years of follow-up providing further evidence for the safety of RDN at long-term. Except for one case of vasospasm during RDN, there were no periprocedural complications documented. A total of 3 renal artery re-interventions were observed (progression of a pre-existing renal artery stenosis, *n* = 2; new-onset renal artery stenosis, *n* = 1). Of note, vascular segments with atherosclerotic plaques were not treated with RF-ablation. Hence, the progression of the pre-existing renal artery stenosis was unlikely related to the RDN procedure.

### Limitations

Several limitations of our study must be discussed. Firstly, this is a single-arm, single-center study without a control group. Secondly, adherence and pill-burden to antihypertensive medication were not examined [38]. Alterations in adherence might have affected the BP changes as suggested in previous studies [39]. However, the mean number of antihypertensive drugs remained stable over time. Thirdly, this study was not a priori powered but designed as a prospective long-term study. Hence, all results should be regarded as hypothesis-generating. Fourthly, in both the Global Symplicity Registry [14] and the present study, the mono-electrode Symplicity Flex RDN catheter system was used with a comparable number of ablations. Whether the same long-term outcomes can also be expected with the other catheter systems remains to be shown.

## Conclusion

In patients with resistant hypertension, RF-RDN was associated with significant and durable reductions in 24-h and office BP through 10 years. RF-RDN was associated with a favorable safety profile and may represent an attractive alternative treatment in certain patients with resistant hypertension.

## Supplementary Information

Below is the link to the electronic supplementary material.

Supplementary file1 (DOCX 143 KB)

## Abbreviations and symbols

ABP: Ambulatory blood pressure
BP: Blood pressure
CKD: Chronic kidney disease
DBP: Diastolic blood pressure
eGFR: Estimated glomerular filtration rate
RDN: Renal denervation
RF: Radiofrequency
RRI: Renal resistive index
SBP: Systolic blood pressure
24-h: 24-Hour

## Declarations

### Declaration of dual publication

We have previously published results of the change in 24-h blood pressure (BP) and renal functions over time as well as limited safety data in a research letter in JACC under the title '10-Year Outcomes of Catheter-Based Renal Denervation in Patients With Resistant Hypertension' (JACC VOL. 81, NO.5, 2023, ISSN 0735-1097). Herein, we present the full data set including different BP measurement methods, BP control rates, utilization of antihypertensive drugs, and changes in renal resistance indices, which are important to assess the efficacy and safety of renal denervation in the long term. The present manuscript therefore differs significantly from the research letter not only regarding the type but also the content of the manuscript, which is novel.

## References

[1] Whelton PK, Carey RM, Aronow WS, Casey DE, Collins KJ, Dennison Himmelfarb C et al (2018) 2017 ACC/AHA/AAPA/ABC/ACPM/AGS/APhA/ASH/ASPC/NMA/PCNA guideline for the prevention, detection, evaluation, and management of high blood pressure in adults. Circ 138:e484–e594. 10.1161/CIR.0000000000000596 10.1161/CIR.0000000000000596 30354654

[2] Williams B, Mancia G, Spiering W, Rosei EA, Azizi M, Burnier M et al (2018) 2018 ESC/ESH guidelines for the management of arterial hypertension: the task force for the management of arterial hypertension of the European Society of Hypertension (ESH) and of the European Society of Cardiology (ESC). Eur Heart J 39:3021–3104. 10.1093/eurheartj/ehy339 30165516 10.1093/eurheartj/ehy339

[3] Egan BM, Li J, Sutherland SE, Rakotz MK, Wozniak GD (2021) Hypertension control in the United States 2009 to 2018: factors underlying falling control rates during 2015 to 2018 across age- and race-ethnicity groups. Hypertension 78:578–587. 10.1161/HYPERTENSIONAHA.120.16418 34120453 10.1161/HYPERTENSIONAHA.120.16418

[4] Johns EJ, Kopp UC, Dibona GF (2011) Neural control of renal function. Compr Physiol 1:731–767. 10.1002/cphy.c100043 23737201 10.1002/cphy.c100043

[5] Lauder L, Azizi M, Kirtane AJ, Böhm M, Mahfoud F (2020) Device-based therapies for arterial hypertension. Nat Rev Cardiol 17:614–628. 10.1038/s41569-020-0364-1 32286512 10.1038/s41569-020-0364-1

[6] Böhm M, Kario K, Kandzari DE, Mahfoud F, Weber MA, Schmieder RE et al (2020) Efficacy of catheter-based renal denervation in the absence of antihypertensive medications (SPYRAL HTN-OFF MED Pivotal): a multicentre, randomised, sham-controlled trial. Lancet 395:1444–1451. 10.1016/S0140-6736(20)30554-7 32234534 10.1016/S0140-6736(20)30554-7

[7] Kandzari DE, Böhm M, Mahfoud F, Townsend RR, Weber MA, Pocock S et al (2018) Effect of renal denervation on blood pressure in the presence of antihypertensive drugs: 6-month efficacy and safety results from the SPYRAL HTN-ON MED proof-of-concept randomised trial. Lancet 391:2346–2355. 10.1016/S0140-6736(18)30951-6 29803589 10.1016/S0140-6736(18)30951-6

[8] Azizi M, Schmieder RE, Mahfoud F, Weber MA, Daemen J, Davies J et al (2018) Endovascular ultrasound renal denervation to treat hypertension (RADIANCE-HTN SOLO): a multicentre, international, single-blind, randomised, sham-controlled trial. Lancet 6736:1–11. 10.1016/S0140-6736(18)31082-1 10.1016/S0140-6736(18)31082-1 29803590

[9] Azizi M, Schmieder RE, Mahfoud F, Weber MA, Daemen J, Lobo MD et al (2019) Six-month results of treatment-blinded medication titration for hypertension control after randomization to endovascular ultrasound renal denervation or a sham procedure in the RADIANCE-HTN SOLO trial. CIRC J 139:2542–2553. 10.1161/CIRCULATIONAHA.119.040451 10.1161/CIRCULATIONAHA.119.040451 30880441

[10] Azizi M, Sanghvi K, Saxena M, Gosse P, Reilly JP, Levy T et al (2021) Ultrasound renal denervation for hypertension resistant to a triple medication pill (RADIANCE-HTN TRIO): a randomised, multicentre, single-blind, sham-controlled trial. Lancet 397:2476–2486. 10.1016/S0140-6736(21)00788-1 34010611 10.1016/S0140-6736(21)00788-1

[11] Ahmad Y, Kane C, Arnold AD, Cook CM, Keene D, Shun-Shin M et al (2022) Randomized blinded placebo-controlled trials of renal sympathetic denervation for hypertension: a meta-analysis. Cardiovasc Revasc Med 34:112–118. 10.1016/j.carrev.2021.01.031 33551282 10.1016/j.carrev.2021.01.031 PMC8813172

[12] Syed M, Osman M, Alhamoud H, Saleem M, Munir MB, Kheiri B et al (2021) The state of renal sympathetic denervation for the management of patients with hypertension: a systematic review and meta-analysis. Catheter Cardiovasc Interv 97:E438–E445. 10.1002/ccd.29384 33179863 10.1002/ccd.29384 PMC8381272

[13] Al GH, Götzinger F, Böhm M, Mahfoud F (2022) Arterial hypertension- Clinical trials update 2021. Nutr Metab Cardiovasc Dis 32:21–31. 10.1016/j.numecd.2021.09.007 34690044 10.1016/j.numecd.2021.09.007 PMC8444354

[14] Mahfoud F, Mancia G, Schmieder RE, Ruilope L, Narkiewicz K, Schlaich M et al (2022) Cardiovascular risk reduction after renal denervation according to time in therapeutic systolic blood pressure range. J Am Coll Cardiol 80(20):1871–1880. 10.1016/j.jacc.2022.08.802 36357087 10.1016/j.jacc.2022.08.802

[15] Kario K, Mahfoud F, Kandzari DE, Townsend RR, Weber MA, Schmieder RE et al (2022) Long-term reduction in morning and nighttime blood pressure after renal denervation: 36-month results from SPYRAL HTN-ON MED trial. Hypertens Res 280–8. 10.1038/s41440-022-01042-8 10.1038/s41440-022-01042-8 PMC9747613 36241705

[16] Mahfoud F, Kandzari DE, Kario K, Townsend RR, Weber MA, Schmieder RE et al (2022) Long-term efficacy and safety of renal denervation in the presence of antihypertensive drugs (SPYRAL HTN-ON MED): a randomised, sham-controlled trial. Lancet 399(10333):1401–1410. 10.1016/S0140-6736(22)00455-X 35390320 10.1016/S0140-6736(22)00455-X

[17] Rader F, Kirtane AJ, Wang Y, Daemen J, Lurz P, Sayer J et al (2022) Durability of blood pressure reduction after ultrasound renal denervation: three-year follow-up of the treatment arm of the randomised RADIANCE-HTN SOLO trial. EuroIntervention 18(8):E677–E685. 10.4244/EIJ-D-22-00305 35913759 10.4244/EIJ-D-22-00305 PMC10241283

[18] Al Ghorani H., Kulenthiran S., Recktenwald MJM., Lauder L, Kunz M, Götzinger F et al (2023) 10-Year Outcomes of Catheter-Based Renal Denervation in Patients With Resistant Hypertension. J Am Coll Cardiol 81(5):517–9. 10.1016/j.jacc.2022.11.038 10.1016/j.jacc.2022.11.038 36725181

[19] Levey AS, Stevens LA, Schmid CH, Zhang Y, Castro AF, Feldman HI et al (2009) A new equation to estimate glomerular filtration rate. Ann Intern Med 150:604–612. 10.7326/0003-4819-150-9-200905050-00006 19414839 10.7326/0003-4819-150-9-200905050-00006 PMC2763564

[20] Sesa-Ashton G, Nolde JM, Muente I, Carnagarin R, Lee R, Macefield VG et al (2023) Catheter-based renal denervation: 9-year follow-up data on safety and blood pressure reduction in patients with resistant hypertension. Hypertension 80(4):811–819. 10.1161/HYPERTENSIONAHA.122.20853 36762561 10.1161/HYPERTENSIONAHA.122.20853

[21] Polonia J, Azevedo A, Monte M, Silva JA, Bertoquini S (2017) Annual deterioration of renal function in hypertensive patients with and without diabetes. Vasc Health Risk Manag 13:231–237. 10.2147/VHRM.S135253 28721063 10.2147/VHRM.S135253 PMC5498504

[22] Yang WY, Melgarejo JD, Thijs L, Zhang ZY, Boggia J, Wei FF et al (2019) Association of office and ambulatory blood pressure with mortality and cardiovascular outcomes. JAMA 322:409–420. 10.1001/jama.2019.9811 31386134 10.1001/jama.2019.9811 PMC6822661

[23] Staplin N, Sierra A De, Ruilope LM, Emberson JR, Vinyoles E, Gorostidi M et al (2023) Relationship between clinic and ambulatory blood pressure and mortality : an observational cohort study in 59 124 patients. 6736(23):1–10. 10.1016/S0140-6736(23)00733-X 10.1016/S0140-6736(23)00733-X 37156250

[24] Weber MA, Schmieder RE, Kandzari DE, Townsend RR, Mahfoud F, Tsioufis K et al (2022) Hypertension urgencies in the SPYRAL HTN-OFF MED Pivotal trial. Clin Res Cardiol 111(11):1269–1275. 10.1007/s00392-022-02064-5 35852582 10.1007/s00392-022-02064-5 PMC9622517

[25] Booth LC, Nishi EE, Yao ST, Ramchandra R, Lambert GW, Schlaich MP et al (2015) Reinnervation following catheter-based radio-frequency renal denervation. Exp Physiol 100:485–490. 10.1113/expphysiol.2014.079871 25573386 10.1113/expphysiol.2014.079871

[26] Singh RR, McArdle ZM, Iudica M et al (2019) Sustained decrease in blood pressure and reduced anatomical and functional reinnervation of renal nerves in hypertensive sheep 30 months after catheter-based renal denervation. Hypertension 73:718–727. 10.1161/HYPERTENSIONAHA.118.12250 30661475 10.1161/HYPERTENSIONAHA.118.12250

[27] Booth LC, Nishi EE, Yao ST, Ramchandra R, Lambert GW, Schlaich MP et al (2015) Reinnervation of renal afferent and efferent nerves at 5.5 and 11 months after catheter-based radiofrequency renal denervation in sheep. Hypertension 65:393–400. 10.1161/HYPERTENSIONAHA.114.04176 10.1161/HYPERTENSIONAHA.114.04176 25403610

[28] Krum H, Schlaich MP, Sobotka PA, Böhm M, Mahfoud F, Rocha-Singh K et al (2014) Percutaneous renal denervation in patients with treatment-resistant hypertension: final 3-year report of the Symplicity HTN-1 study. Lancet 383:622–629. 10.1016/S0140-6736(13)62192- 24210779 10.1016/S0140-6736(13)62192-3

[29] Mahfoud F, Renkin J, Sievert H, Bertog S, Ewen S, Böhm M et al (2020) Alcohol-mediated renal denervation using the peregrine system infusion catheter for treatment of hypertension. JACC Cardiovasc Interv 13:471–484. 10.1016/j.jcin.2019.10.048 32081241 10.1016/j.jcin.2019.10.048

[30] Townsend RR, Walton A, Hettrick DA, Hickey GL, Weil J, Sharp ASP et al (2020) Review and meta-analysis of renal artery damage following percutaneous renal denervation with radiofrequency renal artery ablation. EuroIntervention 16:89–96 32038027 10.4244/EIJ-D-19-00902

[31] Mahfoud F, Böhm M, Schmieder R, Narkiewicz K, Ewen S, Ruilope L et al (2019) Effects of renal denervation on kidney function and long-term outcomes: 3-year follow-up from the Global SYMPLICITY Registry. Eur Heart J 40:3474–3482. 10.1093/eurheartj/ehz118 30907413 10.1093/eurheartj/ehz118 PMC6837160

[32] Vupputuri S, Batuman V, Muntner P, Bazzano LA, Lefante JJ, Whelton PK et al (2003) Effect of blood pressure on early decline in kidney function among hypertensive men. Hypertension 42:1144–1149. 10.1161/01.HYP.0000101695.56635.31 14597644 10.1161/01.HYP.0000101695.56635.31

[33] Chowdhury EK, Langham RG, Ademi Z, Owen A, Krum H, Wing LMH et al (2015) Rate of change in renal function and mortality in elderly treated hypertensive patients. Clin J Am Soc Nephrol 10:1154–1161. 10.2215/CJN.07370714 25901093 10.2215/CJN.07370714 PMC4491290

[34] Zoppini G, Targher G, Chonchol M, Ortalda V, Negri C, Stoico V et al (2012) Predictors of estimated GFR decline in patients with type 2 diabetes and preserved kidney function. Clin J Am Soc Nephrol 7:401–408. 10.2215/CJN.07650711 22282481 10.2215/CJN.07650711

[35] Bakris GL, Williams M, Dworkin L, Elliott WJ, Epstein M, Toto R et al (2000) Preserving renal function in adults with hypertension and diabetes: A consensus approach. Am J Kidney Dis 36:646–661. 10.1053/ajkd.2000.16225 10977801 10.1053/ajkd.2000.16225

[36] Pontremoli R, Viazzi F, Martinoli C, Ravera M, Nicolella C, Berruti V et al (1999) Increased renal resistive index in patients with essential hypertension: a marker of target organ damage. Nephrol Dial Transplant 14(2):360–365. 10.1093/ndt/14.2.360 10069189 10.1093/ndt/14.2.360

[37] Mahfoud F, Cremers B, Janker J, Link B, Vonend O, Ukena C et al (2012) Renal hemodynamics and renal function after catheter-based renal sympathetic denervation in patients with resistant hypertension. Hypertension 60(2):419–424. 10.1161/HYPERTENSIONAHA.112.193870 22733462 10.1161/HYPERTENSIONAHA.112.193870

[38] Böhm M, Townsend RR, Kario K, Kandzari D, Mahfoud F, Weber MA et al (2020) Rationale and design of two randomized sham-controlled trials of catheter-based renal denervation in subjects with uncontrolled hypertension in the absence (SPYRAL HTN-OFF MED Pivotal) and presence (SPYRAL HTN-ON MED Expansion) of antihypertensive medicat. Clin Res Cardiol 109:289–302. 10.1007/s00392-020-01595-z 32034481 10.1007/s00392-020-01595-z PMC7042193

[39] Ewen S, Meyer MR, Cremers B, Laufs U, Helfer AG, Linz D et al (2015) Blood pressure reductions following catheter-based renal denervation are not related to improvements in adherence to antihypertensive drugs measured by urine/plasma toxicological analysis. Clin Res Cardiol 104:1097–1105. 10.1007/s00392-015-0905-5 26306594 10.1007/s00392-015-0905-5

