# Supplementary figures and images for: Ultra-long-term efficacy and safety of catheter-based renal denervation in resistant hypertension: 10-year follow-up outcomes
Source: Clin Res Cardiol. 2024 Mar 7;113(10):1384–92. doi: 10.1007/s00392-024-02417-2 (PMC11420252; doi:10.1007/s00392-024-02417-2)

**Supplementary material**


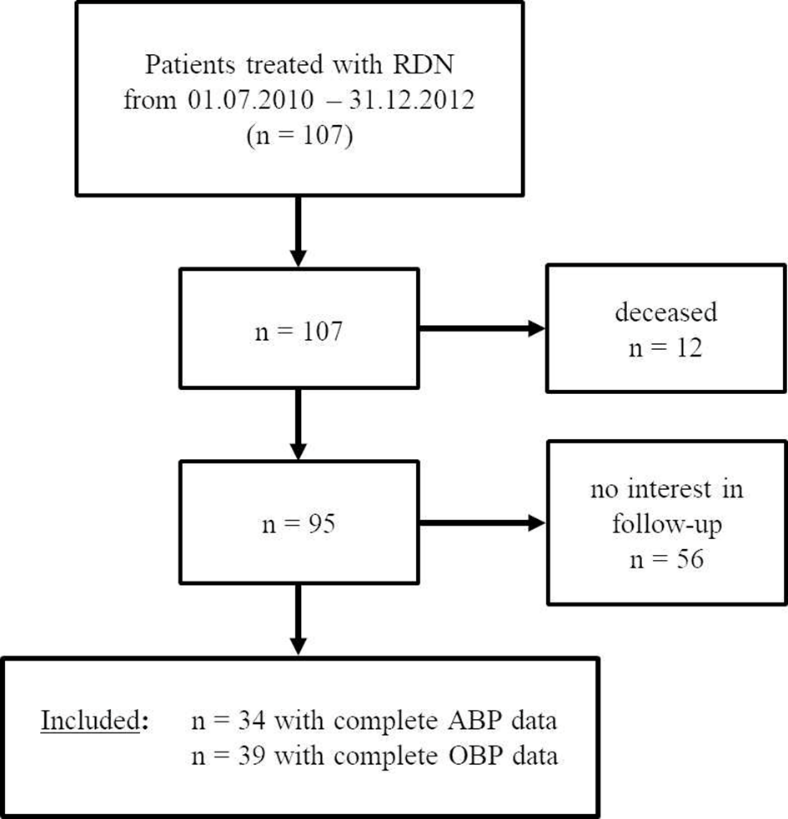


Flow chart of the study.

Supplement: Supplementary file 1 — Supplementary file1 (DOCX 143 KB) [file 392_2024_2417_MOESM1_ESM.docx]
